# Smartphone video games effectively improve cognitive function in middle-aged and elderly patients with chronic schizophrenia: a randomized clinical trial

**DOI:** 10.1038/s41398-025-03364-w

**Published:** 2025-04-18

**Authors:** Miaomiao Zhang, Xinyu Chen, Yitan Yao, Wenhui Wang, Yongjie Zhong, Shengya Shi, Kai Zhang

**Affiliations:** 1 Department of Psychiatry, Chaohu Hospital of Anhui Medical University, Hefei, China; 2 Anhui Psychiatric Center, Hefei, China; 3 School of Mental Health and Psychological Sciences, Anhui Medical University, Hefei, China; 4 Wuxi Mental Health Center, Wuxi, China; 5 Anhui Provincial Key Laboratory for Brain Bank Construction and Resource Utilization, Hefei, China; 6 Department of Psychiatry, The Fourth Affiliated Hospital of Anhui Medical University, Chaohu, Hefei, China

**Keywords:** Schizophrenia, Predictive markers

## Abstract

Cognitive dysfunction in chronic schizophrenia has a serious impact on the quality of life of middle-aged and elderly patients. It is urgent to find an economical and effective treatment to improve cognitive function. The purpose of this study was to explore the effect of free video games on cognitive function and blood factors in middle-aged and elderly patients with chronic schizophrenia. The study began in July 2021 and ended in February 2022. The subjects suffered from chronic schizophrenia and were aged between 40–65 years old. Participants were randomly divided into control group (*n* = 15) and game group (*n* = 12). The control group watched TV for 1 h every day, and the game group played video games for 1 h every day. Both groups were five days a week for six weeks. This study found that compared with the control group, the cognitive function of the game group was significantly improved (*P* < 0.001). The level of GDNF in the game group was significantly increased, and the levels of Tau, MIP-1 and MIP-4 were decreased. Serum GDNF and MIP-1 were significantly correlated with cognitive function. In conclusion, smartphone free video games effectively improve the cognitive function of middle-aged and elderly patients with chronic schizophrenia. In addition, blood factors GDNF, Tau, MIP-1 and MIP-4 may be serum markers for predicting cognitive function. It provides a new idea for the clinical treatment of cognitive impairment. Trial Registration: Chinese Clinical Trial Registry Identifier**:** ChiCTR2100044113

## Introduction

Schizophrenia is one of the most severe and persistent psychiatric disorders, with a lifetime prevalence of approximately 1% [1]. The clinical symptoms of schizophrenia are often manifested as positive symptoms, negative symptoms, cognitive dysfunction, emotional symptoms, excitement and hostility [2]. Chronic schizophrenia refers to the chronic phase after the onset of the disease, characterized by chronic functional disability, negative symptoms, and physical complications [3]. In addition, patients with chronic schizophrenia exhibit a wide range of cognitive impairment. It is well known that cognitive impairment is one of the core symptoms of schizophrenia [4]. And about 98% of schizophrenic patients have cognitive impairment, which seriously affects the quality of life of schizophrenic patients [5]. In addition, cognitive impairment can induce negative symptoms and psychiatric symptoms in patients [6]. Velligan et al. [7] found that cognitive impairment was not related to the severity of psychiatric symptoms, but closely related to dysfunction. Therefore, the improvement of mental symptoms will not improve cognitive impairment. In the long course of the disease, patients with schizophrenia often have cognitive decline with age [8]. Recent studies have shown that the improvement of cognitive function has become an important target for the treatment and prognosis of patients with schizophrenia [9, 10]. Therefore, improving cognitive impairment is an effective way to improve the quality of life of middle-aged and elderly patients with chronic schizophrenia.

At present, the commonly used methods for the treatment of cognitive impairment are antipsychotic drugs, cognitive enhancers and cognitive training [11]. Disappointingly, Nielsen et al. [12] found that antipsychotic drugs have little effect on cognitive impairment in patients with mental classification. A meta-analysis reported that the use of cognitive enhancers was statistically different from the control group, but the effect was not obvious [13]. A study found that patients with schizophrenia treated with cognitive remediation therapy(CRT) had significant improvements in multiple cognitive domains [14]. However, other studies have come to the opposite conclusion. A multicenter randomized controlled clinical trial found that although CRT can improve performance in cognitive training [15]. Nevertheless, compared with the control group, the cognitive function of the cognitive correction treatment group was not significantly improved in the cognitive function test [15]. Obviously, CRT compliance problems may lead to high dropout rates during treatment. Excitingly, recent studies have found that physical activity can improve cognitive function [16]. Nevertheless, the treatment of cognitive impairment in patients with schizophrenia still needs to be explored.

Changes in the activity of neurotrophic factors can regulate the plasticity and activity of nerve cells and maintain the functional and structural integrity of brain neurons [17, 18]. Glial cell-derived neurotrophic factor (GDNF), a member of the transforming growth factor-β superfamily, is a powerful growth factor that can effectively promote the survival of dopaminergic neurons [19, 20]. A decrease in GDNF levels was observed in patients with schizophrenia [21]. Studies in rodents have found that the level of GDNF is related to the cognitive function of possible mice [22]. A clinical study by Marta et al. also suggested that GDNF is associated with cognitive function [23].

Tau is a soluble protein [24]. Several studies have shown that the level of Tau protein is related to cognitive function [25–27]. Alzheimer ‘s disease (AD) is characterized by a progressive decline in cognitive function [28]. Alzheimer ‘s disease is characterized by the accumulation of extracellular β-amyloid (Aβ) plaques and the pathological accumulation of intracellular Tau protein [29]. There are many studies on the relationship between AD and Tau, but there are few studies on cognitive impairment and Tau level in schizophrenia.

Our research group hopes to improve the cognitive function of middle-aged and elderly patients with chronic mental illness through more interesting and simple treatment methods. We observe whether it will have a positive impact on the cognitive function of patients by playing free video games on smartphones in patients with chronic schizophrenia. To explore whether video games have an effect on the levels of blood factors and plasma inflammatory factors, such as the above-mentioned GDNF related to cognitive function, serum total Tau level, macrophage inflammatory protein-1 (MIP-1) and macrophage inflammatory protein-4(MIP-4). We also further evaluated whether blood factors or plasma inflammatory factors can predict the improvement of cognitive function. To the best of our knowledge, we are the first study to evaluate the correlation between serum Tau, MIP-1 and MIP-4 and cognitive function in chronic schizophrenia.

## Methods

### Participants

This study was approved by the Ethics Committee of Chaohu Hospital of Anhui Medical University (ethics committee approval number: 202107-021). The study began in July 2021 and ended in February 2022. All participants were from the Department of Psychiatry, Chaohu Hospital, Anhui Medical University. All participants and their guardians were informed of the study and provided written informed consent.

The inclusion criteria were as follows: (1) aged between 40 and 65 years old, Han nationality; (2) Diagnostic and Statistical Manual of Mental Disorders, Fourth Edition (DSM-IV) Schizophrenia Diagnosis >5 years; (3) Take antipsychotic drugs for at least 8 weeks before enrollment, and the dose of antipsychotic drugs is stable for at least 6 months; (4) Ability to participate in psychopathological assessment.

The exclusion criteria were as follows: (1) DSM-IV diagnosed as obsessive-compulsive disorder or other mental illness; (2) Nervous system diseases; (3) Traumatic brain injury, seizures; (4) Physical examination, electrocardiogram, laboratory tests have obvious clinical abnormalities; (5) pregnancy or breastfeeding; (6) Visual and hearing impairment; (7) Unable to understand and sign informed consent.

### Group and intervention

This study is a single-blind, parallel allocation, randomized clinical trial. The researchers divided the included patients into a control group and a game group through a computer-generated random list. Participants were treated with standard anti-psychiatry for cognitive function. The project lasted for 12 weeks.

The control group watched television for 1 h per day, 5 times a week for 6 weeks. In addition, the game group played video games for 1 h per day, 5 times a week for 6 weeks. We have selected three simple and interesting video games, such as Anipop (Happy Elements Technology), Zuma (PopCap Games), and Talking Tom Gold Run (Outfit7).

### Measures

We used Repeatable Battery for the Assessment of Neuropsychological Status (RBANS) [30], Stroop Color and Word Test (SCWT) [31], Positive and Negative Syndrome Scale (PANSS) [32], Global Assessment of Function (GAF) [33], Patient Health Questionnaire-9 (PHQ-9), General Self-Efficacy Scale (GSES), and Problematic Mobile Game Questionnaire (PMGQ) to assess participants‘ cognitive function, mental symptoms, and mobile phone addiction risk.

All participants underwent repeated measurements at baseline, 3 weeks of treatment, 6 weeks of treatment, 3 weeks after treatment, and 6 weeks after treatment. The evaluators were all consistency-trained psychiatrists, and the evaluators were not aware of the participant grouping at the time of the assessment.

### Blood collection and assays

Peripheral blood was repeatedly collected at baseline, 3 weeks of treatment, 6 weeks of treatment, 3 weeks after treatment, and 6 weeks after treatment. Participants were kept on an empty stomach and peripheral blood was collected at 7:00–7:30am and stored in a 10 ml blood collection tube containing EDTA potassium. At room temperature, the samples were centrifuged at 3500 rpm for 10 min and then centrifuged for 30 min. Serum was stored at −80 °C until determination. Serum TAU, GDNF, MIP-1 and MIP-4 levels were measured using an enzyme-linked immunosorbent assay (ELISA) kits purchased from Bio-Source, Inc.

### Statistical analysis

Statistical analysis was performed using the Statistical Package for Social Sciences Version 27 (SPSS, Chicago, Illinois, USA). The measurement data were expressed as mean ± standard deviation (S.D.). Demographic and clinical data were analyzed by descriptive statistics. Student ‘s t test was used to analyze the numerical variables between groups. Chi-square test was used to analyze categorical variables. Repeated measures analysis of variance was used to analyze the recorded data, followed by post-hoc analysis of the least significant difference. The Pearson correlation coefficient was used to evaluate the correlation between blood factor levels and RBANS. The difference was statistically significant (*P* < 0.05).

## Results

### Demographic data and disease characteristics

**Fig. 1:**
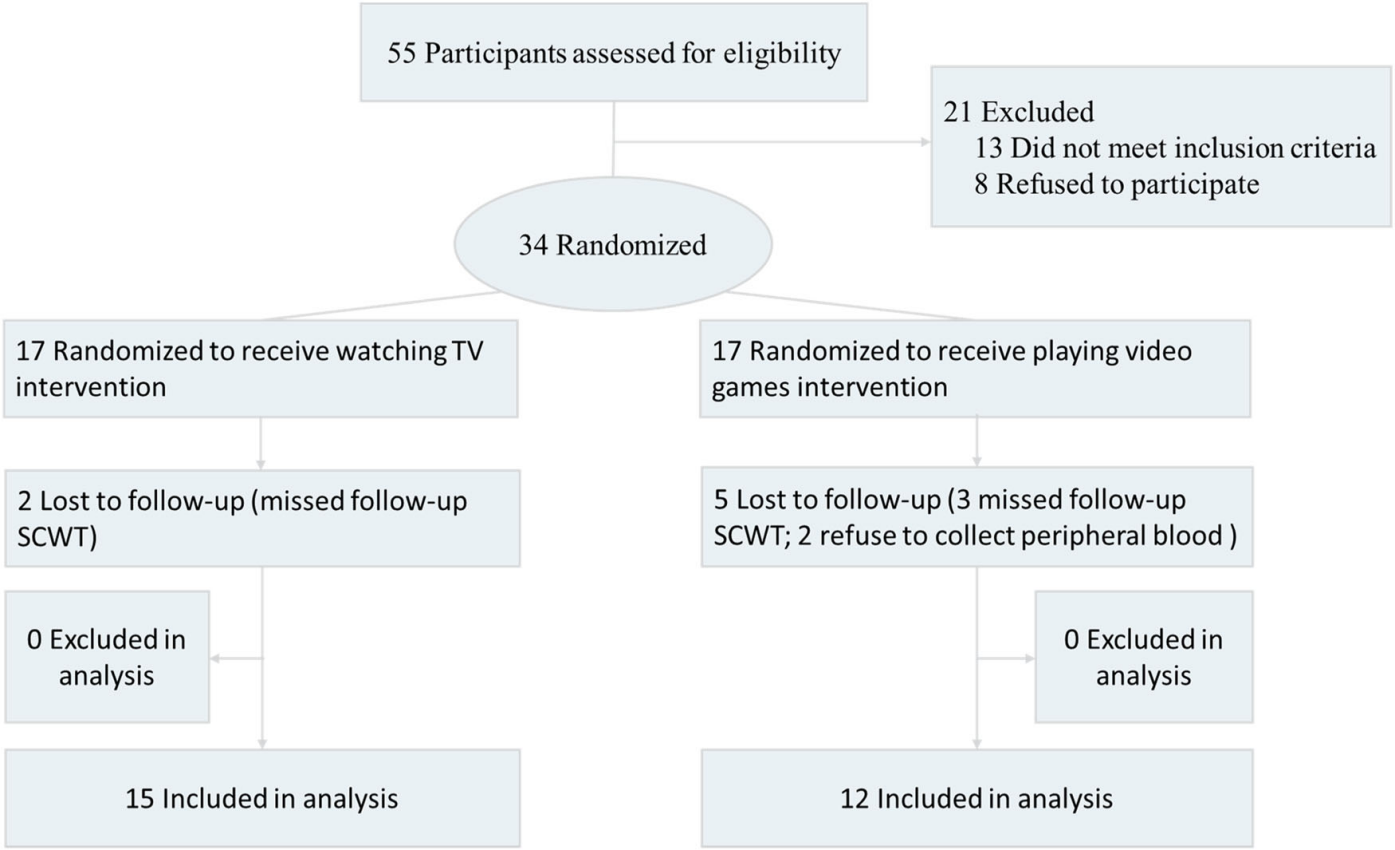
CONSORT flow diagram.

A total of 34 participants were included and randomly assigned to the control group and the game group, with 17 participants in each group. The number of people participating in the whole project in the control group and the game group was 15 and 12 respectively (Fig. 1). There was no statistical difference in basic demographic data between the control group and the game group (Table 1). For example, gender, age, height, weight, body mass index (BMI) and education level (*P* > 0.05). Additionally, there was no significant difference in the age of first onset between the two groups (*P* > 0.05).

**Table 1:** Comparisons of demographic data and disease characteristics between the two group.

	Control group (*n* = 15)	Game group (*n* = 12)	*P* value
Demographic data			
Age, year	50.93 ± 5.958	50.42 ± 6.788	0.835
Sex, F /M	6/9	5/7	0.093
Education, years	7.67 ± 3.62	7.87 ± 3.39	0.817
BMI, kg/m^2^	24.62 ± 5.67	27.25 ± 2.40	0.147
Height, cm	164.1 ± 9.00	165.7 ± 8.97	0.663
Weight, Kg	66.07 ± 15.40	74.83 ± 9.48	0.097
Disease characteristics			
Duration of illness, years	24.68 ± 6.71	30.91 ± 10.23	0.053

*F* Female, *M* Male, *BMI* body mass index. Data are expressed as number for categorical variables mean ± SD for continuous variables.

### Effect of video games on cognitive function

The evaluation results of RBANS are as follows (Table 2). The RBANS test consists of five factors: immediate memory, visuospatial/constructional, language, attention and delayed memory. The immediate memory, language, delayed memory and RBANS total scores of the control group and the game group showed significant improvement over time (*P* < 0.001, *P* = 0.005, *P* < 0.001, *P* < 0.001) (Table 2). In addition, compared with the control group, the delayed memory score and RBANS total score of the game group were significantly improved (*P* = 0.008, *P* = 0,03).

**Table 2:** Comparison of RBANS, SWTC and disease characteristics assessment between the control group and the game group at different time points.

	Control group (*n* = 15)	Game group (*n* = 12)	F_time_	P_time_	F_group_	P_group_	F_time×group_	P_time×group_
RBANS Total score			9.787	<0.001	5.275	0.030	3.156	0.039
Baseline	294.20 ± 48.43	333.25 ± 67.38						
3week	304.07 ± 58.83	351.08 ± 67.28						
6week^a^	310.07 ± 61.05	384.93 ± 78.42						
9week^b^	307.27 ± 65.74	378.08 ± 83.63						
12week	309.20 ± 69.81	365.58 ± 78.15						
RBANS immediate memory			10.600	<0.001	1.754	0.197	0.212	0.761
Baseline	47.40 ± 8.24	55.00 ± 12.20						
3week	54.53 ± 14.24	64.50 ± 16.33						
6week	59.13 ± 19.71	69.67 ± 19.78						
9week	59.73 ± 21.36	67.33 ± 20.63						
12week	60.93 ± 20.24	67.92 ± 21.68						
RBANS Visuospatial/constructional			1.213	0.310	5.301	0.030	2.302	0.082
Baseline	66.73 ± 12.91	71.58 ± 14.90						
3week^b^	64.87 ± 7.91	74.83 ± 16.02						
6week^a^	63.60 ± 8.70	78.92 ± 12.26						
9week^b^	63.73 ± 8.40	75.67 ± 17.50						
12week	62.93 ± 9.90	71.83 ± 14.99						
RBANS language			4.914	0.005	1.741	0.199	2.978	0.033
Baseline	62.73 ± 12.71	65.33 ± 14.93						
3week	63.27 ± 16.40	65.33 ± 14.99^b^						
6week	64.80 ± 17.83	77.00 ± 13.63						
9week	64.33 ± 20.73	78.58 ± 14.60^b^						
12week	64.60 ± 17.85	72.33 ± 15.34						
RBANS attention			0.313	0.734	3.490	0.074	0.743	0.565
Baseline	71.20 ± 17.00	79.08 ± 16.45						
3week	69.47 ± 1943	81.05 ± 13.27						
6week	70.73 ± 16.80	83.08 ± 15.75						
9week	70.20 ± 17.17	81.67 ± 18.34						
12week^b^	69.27 ± 15.71	82.42 ± 16.72						
RBANS delayed memory			6.423	<0.001	8.185	0.008	3.169	0.017
Baseline^b^	46.13 ± 10.01	62.25 ± 22.00						
3week	51.93 ± 18.32	64.92 ± 21.11						
6week^a^	51.80 ± 17.22	76.25 ± 24.26						
9week^a^	60.63 ± 11.45	74.83 ± 22.80						
12week^b^	51.47 ± 18.15	71.83 ± 22.31						
SCWT one word error			2.220	0.072	1.098	0.305	0.581	0.542
Baseline	0.80 ± 1.26	1.75 ± 3.79						
3week	0.33 ± 1.05	1.00 ± 2.30						
6week	0.33 ± 1.05	1.33 ± 3.70						
9week	0.27 ± 1.03	0.42 ± 0.90						
12week	0.40 ± 1.12	1.25 ± 2.56						
SCWT one word time			1942	0.150	0.001	0.974	5.095	0.008
Baseline	25.53 ± 11.17	34.25 ± 27.77						
3week	26.47 ± 10.60	29.92 ± 22.50						
6week	28.13 ± 13.16	23.75 ± 14.69						
9week	26.33 ± 12.59	24.75 ± 14.37						
12week	29.13 ± 18.40	29.92 ± 12.67						
SCWT one color error			0.702	0.533	0.197	0.661	0.948	0.412
Baseline	2.13 ± 3.56	2.00 ± 2.59						
3week	1.80 ± 2.68	1.17 ± 2.62						
6week	2.27 ± 3.31	0.75 ± 1.48						
9week	2.20 ± 4.09	1.83 ± 3.54						
12week	1.80 ± 3.61	2.00 ± 3.71						
SCWT one color time			1.913	0.151	0.701	0.410	1.263	0.293
Baseline	40.73 ± 16.65	38.75 ± 16.01						
3week	39.20 ± 15.42	36.25 ± 13.31						
6week	39.93 ± 15.89	32.89 ± 14.10						
9week	38.73 ± 15.31	34.25 ± 14.28						
12week	40.07 ± 16.60	33.08 ± 13.47						
SCWT two words error			2.162	0.079	0.971	0334	2.934	0.060
Baseline	0.60 ± 0.91	3.25 ± 4.85						
3week	0.47 ± 0.74	1.17 ± 2.72						
6week	1.20 ± 1.26	1.41 ± 2.75						
9week	1.33 ± 1.39	1.41 ± 2.50						
12week	1.47 ± 3.38	2.17 ± 3.95						
SCWT two words time			1.038	0.391	0.161	0.691	3.089	0.019
Baseline	28.73 ± 10.77	41.00 ± 29.15						
3week	29.20 ± 10.40	34.25 ± 26.95						
6week	34.60 ± 10.55	32.83 ± 18.31						
9week	36.60 ± 18.81	36.42 ± 26.82						
12week	35.00 ± 15.92	33.33 ± 20.15						
SCWT two colors error			2.636	0.055	0.792	0.382	0.182	0.910
Baseline	8.33 ± 7.88	7.45 ± 5.00						
3week	7.60 ± 7.44	5.67 ± 5.10						
6week	7.20 ± 6.87	4.75 ± 6.11						
9week	5.93 ± 4.51	4.00 ± 3.74						
12week	7.00 ± 5.81	5.25 ± 5.62						
SCWT two colors time			1.716	0.175	0.365	0.511	1.714	0.715
Baseline	70.40 ± 20.99	63.41 ± 17.16						
3week	60.00 ± 17.94	62.58 ± 16.31						
6week	63.47 ± 16.94	61.50 ± 14.56						
9week	65.67 ± 22.61	60.67 ± 15.70						
12week	66.20 ± 63.90	57.67 ± 15.50						
PANSS Total Score			5.317	0.005	2.896	0.101	3.456	0.030
Baseline	65.87 ± 11.14	60.33 ± 13.60						
3week	66.07 ± 15.29	57.58 ± 15.08						
6week	64.93 ± 14.17	54.83 ± 13.99						
9week	65.27 ± 14.85	54.75 ± 14.26						
12week	65.80 ± 14.91	54.50 ± 14.59						
PANSS Positive Scale			3.771	0.023	0.341	0.564	0.926	0.414
Baseline	14.60 ± 4.77	14.00 ± 4.80						
3week	14.33 ± 5.64	13.25 ± 5.22						
6week	14.27 ± 5.20	12.91 ± 5.05						
12week	14.13 ± 5.26	12.75 ± 5.15						
PANSS Negative Scale			3.139	0.043	8.341	0.008	3.074	0.046
Baseline^b^	19.60 ± 4.76	15.42 ± 4.78						
3week^b^	19.60 ± 4,85	15.00 ± 6.19						
6week^a^	19.33 ± 4.98	13.41 ± 4.44						
9week^a^	19.60 ± 5.46	13.25 ± 4.63						
12week^a^	19.87 ± 5.66	13.50 ± 4.72						
PANSS General Psychopathology Scale			2.516	0.090	0.976	0.333	1.937	0.154
Baseline	31.67 ± 4.44	31.17 ± 6.13						
3week	32.13 ± 7.67	29.58 ± 5.92						
6week	31.33 ± 5.95	28.75 ± 6.22						
9week	31.40 ± 6.56	28.83 ± 6.37						
12week	31.80 ± 6.35	28.50 ± 6.48						
PHQ-9 ^d^			5.251	0.008	1.165	0.291	0.642	0.533
Baseline	5.53 ± 5.90	4.91 ± 4.10						
3week	6.47 ± 7.45	4.42 ± 4.32^b^						
6week	4.20 ± 5.99	1.75 ± 1.66^b^						
9week	4.27 ± 5.03	1.83 ± 1.40						
12week	3.67 ± 4.06	2.75 ± 1.81						
GAF			6.774	0.006	0820	0.374	7.558	0.003
Baseline	60.67 ± 12.08	61.33 ± 12.39						
3week	60.47 ± 11.70	63.83 ± 12.00						
6week	61.33 ± 11.24	65.08 ± 11.67						
9week	60.87 ± 11.84	66.41 ± 11.72						
12week	60.07 ± 12.50	67.08 ± 11.65						
GSE			1.148	0.335	0.068	0.796	0.583	0.625
Baseline	23.67 ± 9.85	23.00 ± 4.26						
3week	23.47 ± 7.30	24.17 ± 3.24						
6week	23.27 ± 7.66	24.67 ± 5.85						
9week	24.53 ± 8.54	24.25 ± 4.85						
12week	23.53 ± 9.13	24.75 ± 4.77						
PMGQ			1.407	0252	4.212	0.051	0.839	0.460
Baseline	4.20 ± 0.56	4.42 ± 0.79						
3week	4.47 ± 0.91	5.33 ± 1.78						
6week	4.26 ± 0.59	5.50 ± 2.75						
9week	4.46 ± 1.06	5.67 ± 2.87						
12week	4.26 ± 1.03	5.58 ± 2.71						

*RBANS* repeatable battery for the assessment of neuropsychological status, *PANSS* positive and negative syndrome scale, *PHQ-9* patient health questionnaire-9, *GAF* global assessment of functioning, *GSE* general self-efficacy scale, *PMGQ* problematic mobile gaming questionnaire.

^a^Comprise with control group *P* < 0.01 after Post Hoc analysis.

^b^Comprise with control group *P* < 0.05 after Post Hoc analysis.

The scoring results of SCWT are as follows (Table 2). There was no significant difference in the group effect between the control group and the game group (all *P* > 0.05) (Table 2). And there was no significant difference between the two groups (all *P* > 0.05) (Table 2). However, the interaction effect (time×group) between SCWT one word time and SCWT two words time was statistically different (F_time×group_ = 5.095, P_time×group_ = 0.008; F_time×group_ = 3.089, P_time×group_ = 0.019) (Table 3).

**Table 3:** Comparisons of GDNF, Tau, MIP-1 and MIP-4 levels between control group and game group at different timepoint.

	Control group (*n* = 15)	Game group (*n* = 12)	F_time_	P_time_	F_group_	P_group_	F_time×group_	P_time×group_
GDNF			2.503	0.047	161.477	<0.001	1.750	0.145
Baseline^a^	716.55 ± 118.98	365.32 ± 145.73						
3week^a^	722.63 ± 124.86	466.85 ± 162.32						
6week^a^	731.96 ± 100.47	519.00 ± 149.34^b^						
9week^a^	804.05 ± 91.19^b^	459.98 ± 131.25						
12week^a^	680.15 ± 123.95	451.20 ± 123.95						
Tau			4.856	0.001	136.338	<0.001	8.808	<0.001
Baseline^a^	102.89 ± 28.80	207.87 ± 26.83						
3week^a^	99.84 ± 21.35	170.51 ± 29.81^a^						
6week^a^	115.14 ± 21.82	166.75 ± 32.70^a^						
9week^a^	113.97 ± 27.31	142.20 ± 27.03^a^						
12week^a^	106.70 ± 21.68	156.71 ± 21.81^a^						
MIP-1			10.002	<0.001	87.999	<0.001	4.238	0.003
Baseline^a^	108.96 ± 24.83	183.66 ± 25.90						
3week^a^	108.92 ± 24.97	174.11 ± 22.20						
6week^a^	101.55 ± 23.22	149.98 ± 28.77^b^						
9week^a^	91.87 ± 21.05	134.45 ± 30.42^a^						
12week^a^	102.93 ± 31.25	127.63 ± 27.16^a^						
MIP-4			4.353	0.003	201.905	<0.001	3.556	0.009
Baseline^a^	65.90 ± 16.03	110.20 ± 15.66						
3week^a^	71.35 ± 14.30	98.03 ± 15.74^a^						
6week^a^	62.63 ± 14.71	89.79 ± 13.13^a^						
9week^a^	66.00 ± 14.00	84.82 ± 14.26^a^						
12week^a^	67.00 ± 11.07	84.04 ± 10.63^a^						

*GDNF* glial cell line-derived neurotrophic factor, *Tau* tau protein, *MIP-1* macrophage inflammatory factor-1, *MIP-4* macrophage inflammatory factor-4.

^a^Comprise with control group *P* < 0.01 after Post Hoc analysis.

^b^Comprise with control group *P* < 0.05 after Post Hoc analysis.

### Effect of video games on mental symptoms

The PANSS score results showed that the PANSS total score, positive symptoms and negative symptoms had significant time effects (all *P* < 0.05) (Table 2). Moreover, we observed that the negative symptoms of the game group were significantly improved (*P* = 0.008) (Table 2). The GAF evaluation results of both groups had a significant time effect (*P* < 0.05) (Table 2). The scores of PHQ-9 showed that the participants in the control group and the game group had a significant improvement in mood over time (*P* < 0.05). However, neither GAF nor PHQ-9 had a significant group effect (all *P* > 0.05) (Table 2). What’s more, there was no significant difference in GSES scores between the two groups (*P* > 0.05) (Table 2). The PMGQ scores of the control group and the game group were lower than the critical score at all time points (critical score = 8).

### Effects of video games on TAU, GDNF, MIP1 and MIP4

The level of TAU in serum had a significant time effect, and compared with the control group, the level of TAU in the game group decreased significantly (*P* < 0.001). Significant changes in GDNF, MIP1 and MIP4 were observed over time (all *P* < 0.05). The level of GDNF in the game group was significantly increased. Moreover, MIP1 and MIP4 in both groups had significant group effects (F_time×group_ = 4.238, P_time×group_ = 0.003; F_time×group_ = 3.556, P_time×group_ = 0.009).

### Correlation between GDNF and cognitive function

**Fig. 2:**
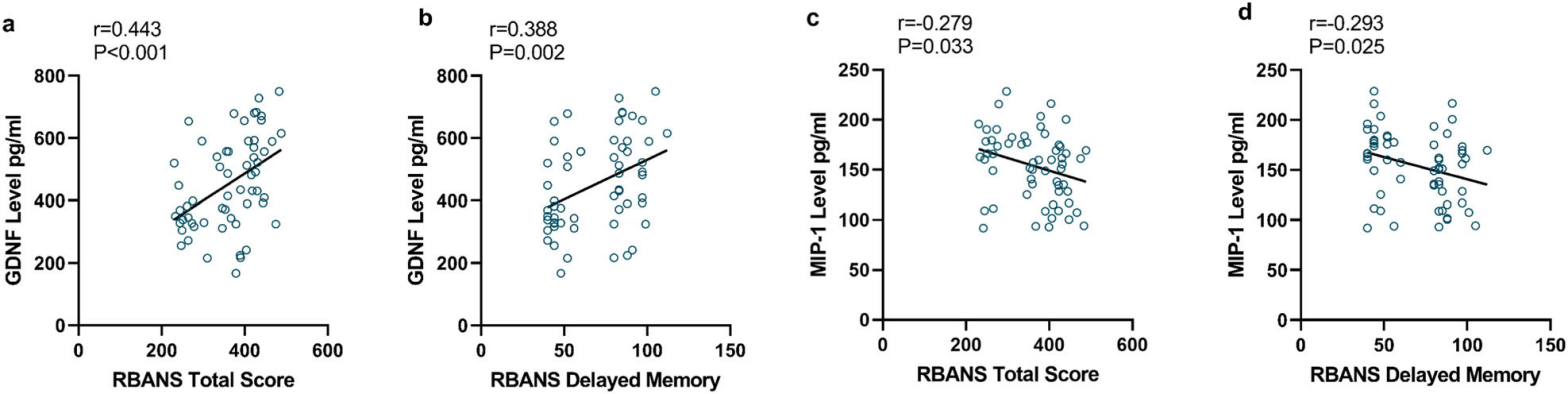
Correlation between blood factors and cognitive function. **a** The correlation between RBANS total score and the level of GDNF; **b** The correlation between RBANS Delayed Memory score and the level of GDNF; **c** The correlation between RBANS total score and the level of MIP-1; **d** The correlation between RBANS Delayed Memory score and the level of MIP-1.

We further analyze the correlation and try to find out some correlations (Fig. 2). Interestingly, we found a significant positive correlation between serum GDNF level and RBANS total score (r = 0.442, *P* < 0.001) (Fig. 2). Moreover, with the increase of GDNF level, the delayed memory score increased (r = 0.388, *P* = 0.002) (Fig. 2).

### Correlation between MIP-1 and cognitive function

It is worth noting that there is a significant negative correlation between the level of MIP-1 in the game group and the total score of RBANS for assessing cognitive function (r = −0.279, *P* = 0.033) (Fig. 2). Moreover, there was a significant negative correlation between the MIP-1 level and the score of delayed memory in the game group (r = −0.293, *P* = 0.025) (Fig. 2).

## Discussion

Our research shows that video games can effectively improve the cognitive impairment of middle-aged and elderly patients with chronic schizophrenia. During the 6-week treatment period, the overall cognitive function of the game group was significantly improved, especially the delayed memory. In addition, the negative symptoms of the game group also improved a lot. And the treatment effect has a certain continuity. After 6 weeks of treatment, the overall cognitive function and negative symptoms of the game group were still significantly improved. The serum results of this experiment showed that video games can increase the level of serum GDNF in middle-aged and elderly patients with chronic schizophrenia, and decrease the levels of Tau, MIP-1 and MIP-4.

So far, the U.S. Food and Drug Administration or China’s National Drug Administration has not approved any drugs for the treatment of cognitive deficits in patients with schizophrenia [34]. Many studies have focused on cognitive enhancement drugs and cognitive training. A meta-analysis comparing the efficacy of different types of cognitive enhancers in patients with schizophrenia shows that cognitive-enhancing drugs can indeed bring some improvement in cognitive impairment, but the effect is very small. There is also a risk of side effects [13]. Iwata et al. [35] indicated that glutamate positive modulators did not significantly improve the cognitive function of patients with schizophrenia. For the cognitive function of patients with chronic schizophrenia, CRT is a very effective treatment method under the premise of seriously completing the rehabilitation plan, especially in combination with mental rehabilitation [16].

Unfortunately, not everyone can be conditioned to receive CRT. In the United States, about 50% of patients with schizophrenia do not receive any treatment [36]. Previous studies have shown that commercial video games can improve the cognitive function of elderly patients with schizophrenia [37]. In addition, our results demonstrate that free video games can also improve the cognitive function of middle-aged and elderly patients with chronic schizophrenia. Roberts et al. [38] believed that there was no significant difference between video games and CRT in improving the function of patients with schizophrenia. Therefore, the results of this study provide a free and interesting way to improve cognitive function for more middle-aged and elderly patients with chronic schizophrenia.

According to research, an important neural mechanism by which video games improve cognitive function is changes in brain plasticity [39]. In particular, some specific brain regions have undergone structural changes. For example, the prefrontal, hippocampus and parietal lobes [40]. The use of video games can increase the volume of gray matter in the brain of healthy people, especially in the hippocampus [41]. Behavioral and magnetic resonance imaging studies of schizophrenia have shown a decrease in cortical gray matter volume and a decrease in hippocampal volume [42]. Recent studies [43] have found that both 3D games and 2D games can enhance the connection between the prefrontal lobe and the hippocampus, further demonstrating that video games can bring beneficial neural structural plasticity to patients with schizophrenia. Aerobic exercise improves cognitive function by improving cardiorespiratory endurance and inducing neural plasticity [44, 45]. Bang-Kittilsen et al. [45] compared the effects of aerobic exercise and video games on schizophrenia. There was no significant difference in the improvement of cognitive function between video games and aerobic exercise. Video games can still induce neuroplasticity without improving cardiopulmonary function. It shows that video games may have a different mechanism of action from aerobic exercise. As observed in this study, video games improve cognitive function may also be achieved by regulating the expression of some biological factors.

As we all know, The cognitive impairment of schizophrenia is related to the abnormal transmission of dopamine in the striatum [46]. GDNF can promote the release and reuptake of dopamine in the striatum. Some studies have shown that the level of GDNF increased after 3 months of treatment in patients with first-episode schizophrenia, which was positively correlated with cognitive function, indicating that GDNF may have neuroprotective effects [23]. Chuanxi et al. [19] found that the level of GDNF increased and the patient’s cognitive function was better. Similarly, our research also proves this statement. Therefore, video game training is likely to cause an increase in the release of GDNF in patients with schizophrenia, thereby alleviating the abnormal transmission of dopamine in the striatum.

The elevated level of total tau protein in cerebrospinal fluid is usually considered to be a sensitive marker of AD neurodegeneration, which is related to the decline of cognitive function in AD [47]. However, Schönknecht et al. [48] found that Tau levels did not increase in the cerebrospinal fluid of patients with schizophrenia. In addition, a recent study has shown that the concentrations of Aβ 1–42 (amyloid brain load) and tau in cerebrospinal fluid of elderly patients with schizophrenia are related to cognitive and structural markers. However, it is inconsistent with neurodegeneration and the level of tau protein in cerebrospinal fluid of elderly patients with schizophrenia is normal. This suggests that the causes of cognitive decline in patients with schizophrenia and AD may not be the same. Schizophrenic patients are due to neurodevelopmental brain damage, and AD is a neurodegenerative disease in the brain [49, 50]. The detection of Tau in cerebrospinal fluid requires lumbar puncture, and it is difficult to apply widely. However, peripheral venous blood sampling is cheaper, faster and less painful for patients. Therefore, we chose to detect the level of Tau protein in serum. After the improvement of cognitive function, the level of serum Tau decreased. This is consistent with a previous study that suggested that elevated plasma total tau can cause neurodegenerative changes of any cause. Higher Tau levels were associated with the risk of cognitive decline and mild cognitive impairment [47]. However, one study, contrary to our conclusion, showed that the levels of serum total Tau and phosphorylated Tau in schizophrenic patients were lower than those in healthy controls [50].

As far as we know, there is no study on the relationship between serum MIP-1 and MIP-4 and cognitive function in middle-aged and elderly patients with schizophrenia. Our study shows that the level of serum decreases after the cognitive function of the game group is effectively improved. And recent studies have shown that the cognitive function of rats after Sophoricoside treatment is improved, and the level of serum MIP-1 is reduced [51]. However, some studies have shown that in AD, cognitive status at baseline is positively correlated with MIP-1β, and MIP-1β is negatively correlated with cognitive decline after 1 year [52]. Pineda et al. [53] said that although the cognitive scale scores of adolescent patients with Neyman-Pick disease type C remained relatively stable, the cognitive ability of patients with premature aging and late aging decreased. Compared with adolescents, patients with premature aging and late aging had higher levels of macrophage active plasma MIP-4. Similarly, Campbell et al. [54] also said that there was a strong negative correlation between cerebrospinal fluid MIP-4 and the age of onset of the nervous system in patients with Neyman-Pick disease type C. Therefore, our findings may provide new serum markers for the study of cognitive function in middle-aged and elderly patients with schizophrenia.

### Limitations

In this study, random grouping was used. There was no difference in basic demographic data and disease symptoms between the two groups. Due to the small sample size of this study, the measured blood factors and baselines of some scales were different, but the results of time effects would not be affected. Our research group will further expand the sample and further explore the treatment and serum markers related to cognitive function.

## Conclusions

In conclusion, our study shows that free smartphone video games can effectively improve the cognitive function of middle-aged and elderly patients with chronic schizophrenia in the process of playing mobile phones in daily life. The level of GDNF in participants with video game training increased, and the levels of Tau, MIP-1 and MIP-4 decreased, which may be used as serum markers to evaluate the cognitive function of patients with schizophrenia. The potential mechanism of how free smartphone video games can improve cognitive function still needs further study.

## Data Availability

The data was used to generate results of this study are available from the corresponding author upon reasonable request.
